# Observations on Hare-Lip

**Published:** 1851-01

**Authors:** Alfred A. Blandy

**Affiliations:** Baltimore.


### ARTICLE VI.

*Observations on Hare-lip.* By ALFRED A. BLANDY, M. D., Baltimore.

HARE-LIP is a congenital deformity, which from its frequent occurrence, would lead one to suppose that its cause could be easily ascertained, but the contrary of this is the case, as the explanations of the credulous and superstitious have been too unreasonable, and approved writers upon this point are almost all silent, leaving curious conjecture to fill the void as fancy may best dictate.

Some have attributed this malformation to the manner of development of the superior maxillary bones; and here again anatomists differ as to the number of points of ossification, some determining upon two, others upon three. The latter would certainly seem the most probable, and to account for phenomena transpiring during development. *This* fact must appear as conclusive, that ossification of the superior maxilla often takes place at three separate points, which upon being interrupted by any cause disturbing the formative economy, fail of natural union, leaving the various descriptions of fissures which are found in the great number of defective palates, and the frequent existence of the disease under description.

This supposition is supported by Gross, in his Pathological Anatomy, where he states that it always depends upon the arrest of the natural development of these parts; but without any distinct division of the points of development. Cruvellier acknowledges that he has often noticed in maxillary bones of the fetus and often of the adult, remarkable fissures, which would seem to indicate the separation of the bone at some time into three pieces, and terms this fissure the *incisive fissure*, visible on each side of the palatine arch, from between the cuspid and incisor teeth back to the anterior palatine canal, circumscribing that portion of the bone which sustains the incisors, and constitutes the intermaxillary of lower animals, and ascribes the seat of *hare-lip* to reside in the solution of continuity of these parts, assuming it as probable that this portion of the maxillary (incisive) is developed from one special point, and this is supported by several anatomists, as Bertin, Bichard and others. Here, then, is a philosophical ground upon which we can determine the cause of this deformity, capable of accounting for all of the various forms in which it is found, viz. simple and complicated hare-lip, fissure of the palate, &c.

Simple separation of the lip, is divided into the natural and the accidental, the former being a defect in formation, the latter the consequence of a wound. Both produce an irregular space of greater or less width, varying from close contact to a very considerable distance, and most frequently involve the front teeth, by their projecting within or without the alveolar arch.

Occasionally, we find even the single cleft and fissure much complicated by a perforation in the palate bones, which are sometimes wanting; again the soft parts are found deficient, the septum nasi distorted in shape, size or position, giving to the mouth and nose a very unsightly aspect.

S. Cooper relates a case, described by Tronchin, in which the *lower lip* was alone affected, and under this condition pictures the most fearful consequences, if unrelieved; from the escape of saliva, imperfect digestion follows, and death most likely ensuing in a short time, if not prevented by removal of the deformity, and thereby its consequences. But as simple fissure of the lip in the superior jaw, is now regarded as a less important matter, and as the closer description of the complicated, will embody all the necessary particulars applicable to the simple, we will pass it by with a few remarks upon the age; and here the divided state of opinion in our highest authorities, as regards the most fitting time for operating, almost absolutely forces each operator to exercise his own judgment in each particular case, as to the time when it is best to be performed. Ledran, B. Bell, Roonhuysen, Lawrence, Mütter, &c., advise an early operation—as early as a few weeks after birth—setting aside the many objections offered, as inconsiderable; and some intimate that failure in operating on the infant is most generally the result of carelessness. Dr. S. P. Hullihen, of Wheeling, Va., in an article in vol. iv, page 244 of the Journal of Dental Science, offers many good reasons for early operation, and to which we gladly refer our readers for valuable information, founded upon much successful experience. M. Louis, Cooper, Chelius, South and others, recommend a much later period, ranging from three months to two years. The more modern practice is that which is followed and recommended by Dr. Hullihen in the before mentioned paper, and perhaps is, as a general thing, the most correct. The instrument most used in operating is the scissors; but of course this is left to the preference of the surgeon; many now prefer the knife, as in the use of even the knife-bladed scissors, there must be a considerable crushing or bruising effect produced before excision is complete; and as there is no objection to the use of the bistoury, answering as it does the most desirable ends, it were safest never to depart from its employment.

Complicated hare-lip is perhaps more irregular in its peculiarities than the simple, being found to consist in forms, all differing from each other, from the mere double fissure of the lip, without any other lesion of surrounding parts, to the combination of the lip, alveoli, maxilla, vomer, palate bones, uvula and the tissues in connection. Most frequently, it involves but a part of these defects, consisting of a single fissure of the palate, with the anterior part of the alveolar border displaced, in protruding with that portion of the lip towards the extremity of the nose. When to this condition is added a second fissure, forming one upon each side of the median line, it becomes the most lamentable, as closing of these fissures cannot be safely attempted until an age in which the patient can be subjected to many restrictions, and to the exercise of much self-command and co-operation. The external appearance can be corrected, but articulation and mastication must remain greatly imperfect until such a condition be removed. Subjoined is a case, occurring under my observation, of considerable novelty, the subject being a little girl of two years and some months of age, who, with this exception, was handsomely developed; but so unsightly was the deformity, that she repulsed every one who approached her, striking them with still greater disgust in her attempts at speech, as, with double fissure of the lip, was double fissure of the palate and division in the uvula, the opening in the palate terminating immediately anterior to this appendage. Between these fissures appeared the portion of maxilla disconnected from either side, and joined only to the palatine bones at its posterior extremity, forming a large septum, as it were, to the united cavities of the nose and mouth, as it was in regular connection with the vomer and septum nasi. At the anterior alveolar border it assumed a cylindrical shape, in continuing its length to very near the extreme point of the nose, where it enlarged into a considerable bulb, and contained the two temporary front incisors, which were ordinarily covered by that portion of the lip having been carried out along with, and in front of the tubercle. This, owing to its large size, greatly impeded the passage of air, in and out of the nostrils, and presented also a serious obstacle to the introduction of food into the mouth, which, by subjecting it to constant irritation, had no doubt increased its size, producing a flexible condition, so that it could be easily moved in its pendulous position, and proving it to be a cartilaginous attachment to the nose. The lip upon either side closely embraced its neck, where the junction of the maxilla should be, so that it swelled out as soon as it passed its anterior surface, giving it length below the lip, and greater breadth upon both sides of each fissure.

I do not remember to have ever seen a rationale of the cause of these projections given by any author, and as they are never to be found at birth in these singular positions, there must be some mechanical cause existing, which produces the great extension so frequently met with. In most cases, I doubt not, it is owing to the manner in which the child uses the nipple, which, under these circumstances, is placed under the tongue, and between it and the lip, in this manner drawing its sustenance only by great force, which force must be supported by the intermaxillary tubercle, as this part is always the most prominent, and the first coming in contact with the tongue. The divided lip being able to give only a small resistance, this, in consequence, is continually being pushed upwards and outwards, and remaining thus subject to this action, until the neighboring parts are forced to bear their proportion of support, which is not the case until the whole is pushed some distance above the lip.

Immediate operation was determined upon, as soon as the patient could be placed in a proper condition ; and for this purpose she was restricted to a moderate diet for one week, with close attention to the bowels, with a view of correcting an irritable and feverish state of the system. On the seventh day, an active saline cathartic was administered, producing a copious evacuation, which, when it had ceased, left the patient in a fine condition for the operation. Upon consultation with a medical friend, I determined to abandon the use of the interrupted suture, for that of the simple, as also the use of the scissors, preferring that of the knife, feeling assured, from the irascible temper of the child, with the difficulty of applying pins in this case, to obtain a permanent juxtaposition, that the operation could not be executed as well, or allowed to remain as undisturbed, with the pins as without them. And the result has induced me to abandon them in all after operations of the kind, and in which determination, I am happy to say, I am not alone. The child was tightly enveloped, by using a long towel passing around both shoulders, and firmly enclosing the arms, continuing the wrapper quite down to the feet, so as to prevent all opposition ; and thus supported by two assistants, with all necessary articles within reach, I made an incision immediately beneath the portion of the lip lying over the tubercle, upwards to the base of the nose, and from thence backwards along the base of the nose, to the junction of the maxilla, thus separating the septum and the tubercle, to a point ranging with the natural arch of the alveolus; then, with the bone forceps, excised the tubercle at its neck, within the lips, leaving the small portion of lip pendulating alone at the point of the nose. In cutting the bone at the neck of the tubercle, a small artery was severed in its substance, the hemorrhage from which was, with difficulty, arrested, as the vessel had receded some distance within the severed edges, and resisted the application of caustics, such as the muriated tincture of iron, sulphuric acid, &c. each failing, I applied tannic acid with no better success; the child began to sink rapidly from the excessive loss of blood, and as a last resort, I compressed the bone with a small pointed pair of pliers, which entirely checked the bleeding, but not until the child had fainted; the syncope lasting for some forty minutes, stimulants were used, which finally produced restoration sufficiently to proceed. The edge of the lip was now pared off downwards, as also the edges of the piece attached to the nose, with a bistoury, cutting upon a hard and flat piece of wood. Fortunately, little hemorrhage followed these incisions, from the great exhaustion previously induced, so that I at once applied the simple suture, by compressing the piece from the nose down to its proper position between the notch in the lip, and united it by two stitches upon each side; then, with a strip of adhesive plaster, reaching from ear to ear, broad at both ends, narrow in the middle, by which the lip was secured in its proper position, and holding the cut surfaces firmly together; then, by inserting a sufficiently large roller upon each cheek, under the strap, the action of the muscles was prevented from straining upon or separating the uniting wounds. In the centre of the strap were several small openings, for the escape of any matter which might otherwise accumulate and cause undue irritation. Then, with a cap bandage, completed the dressing, and with the necessary cautions to the nurse, the patient was left quietly sleeping. Union took place immediately, and in four days the threads were removed without trouble, the plaster again adjusted for four more days, when, with ordinary soft dressings, it healed kindly, leaving but a slight vestige of the operation, the nose being a little depressed; but the age of the patient justified the opinion of its entire recovery from so slight a deformity.

